# Novel drug targets in cell wall biosynthesis exploited by gene disruption in *Pseudomonas aeruginosa*

**DOI:** 10.1371/journal.pone.0186801

**Published:** 2017-10-18

**Authors:** Ayssar A. Elamin, Susanne Steinicke, Wulf Oehlmann, Yvonne Braun, Hanaa Wanas, Eduard A. Shuralev, Carmen Huck, Marko Maringer, Manfred Rohde, Mahavir Singh

**Affiliations:** 1 LIONEX Diagnostics and Therapeutics GmbH, Braunschweig, Germany; 2 Institute of Environmental Sciences, Kazan Federal University, Kazan, Tatarstan, Russian Federation; 3 Central Research Laboratory, Kazan State Medical Academy – Branch Campus of the FSBEI FPE RMACPE MOH Russia, Kazan, Tatarstan, Russian Federation; 4 mfd Diagnostics GmbH, Wendelsheim, Germany; 5 Central Facility for Microscopy, Helmholtz Centre for Infection Research, Braunschweig, Germany; Centre National de la Recherche Scientifique, FRANCE

## Abstract

For clinicians, *Pseudomonas aeruginosa* is a nightmare pathogen that is one of the top three causes of opportunistic human infections. Therapy of *P*. *aeruginosa* infections is complicated due to its natural high intrinsic resistance to antibiotics. Active efflux and decreased uptake of drugs due to cell wall/membrane permeability appear to be important issues in the acquired antibiotic tolerance mechanisms. Bacterial cell wall biosynthesis enzymes have been shown to be essential for pathogenicity of Gram-negative bacteria. However, the role of these targets in virulence has not been identified in *P*. *aeruginosa*. Here, we report knockout (k.o) mutants of six cell wall biosynthesis targets (*murA*, PA4450; *murD*, PA4414; *murF*, PA4416; *ppiB*, PA1793; *rmlA*, PA5163; *waaA*, PA4988) in *P*. *aeruginosa* PAO1, and characterized these in order to find out whether these genes and their products contribute to pathogenicity and virulence of *P*. *aeruginosa*. Except *waaA* k.o, deletion of cell wall biosynthesis targets significantly reduced growth rate in minimal medium compared to the parent strain. The k.o mutants showed exciting changes in cell morphology and colonial architectures. Remarkably, Δ*murF* cells became grossly enlarged. Moreover, the mutants were also attenuated *in vivo* in a mouse infection model except Δ*murF* and Δ*waaA* and proved to be more sensitive to macrophage-mediated killing than the wild-type strain. Interestingly, the deletion of the *murA* gene resulted in loss of virulence activity in mice, and the virulence was restored in a plant model by unknown mechanism. This study demonstrates that cell wall targets contribute significantly to intracellular survival, *in vivo* growth, and pathogenesis of *P*. *aeruginosa*. In conclusion, these findings establish a link between cell wall targets and virulence of *P*. *aeruginosa* and thus may lead to development of novel drugs for the treatment of *P*. *aeruginosa* infection.

## Introduction

*Pseudomonas aeruginosa* is a wide-spread Gram-negative bacterium. It is reported as the third leading cause of nosocomial infections responsible for life-threatening infections of immunocompromised and cystic fibrosis patients and is main cause of hospital-acquired infections, particularly for burns victims [1, 2].

Biofilm forming bacteria like *P*. *aeruginosa* are armed with success-arsenals such as environmental adaption to distinct environments with low nutriments showing a characteristic regulation in gene expression profile [3]. Biofilm formation also leads to a higher antibiotic tolerance and gives the necessary time to acquire antibiotic resistance [3, 4]. Antibiotic resistance mechanisms have been studied extensively in *P*. *aeruginosa*. Common mechanisms/factors are active efflux and/or decreased uptake of drugs (membrane permeability), modification of the drug, oxidative phosphorylation, lipopolysaccharide (LPS) composition, cyclic di-guanosine monophosphate (c-di-GMP) levels and quorum sensing [3–8].

**Fig 1:**
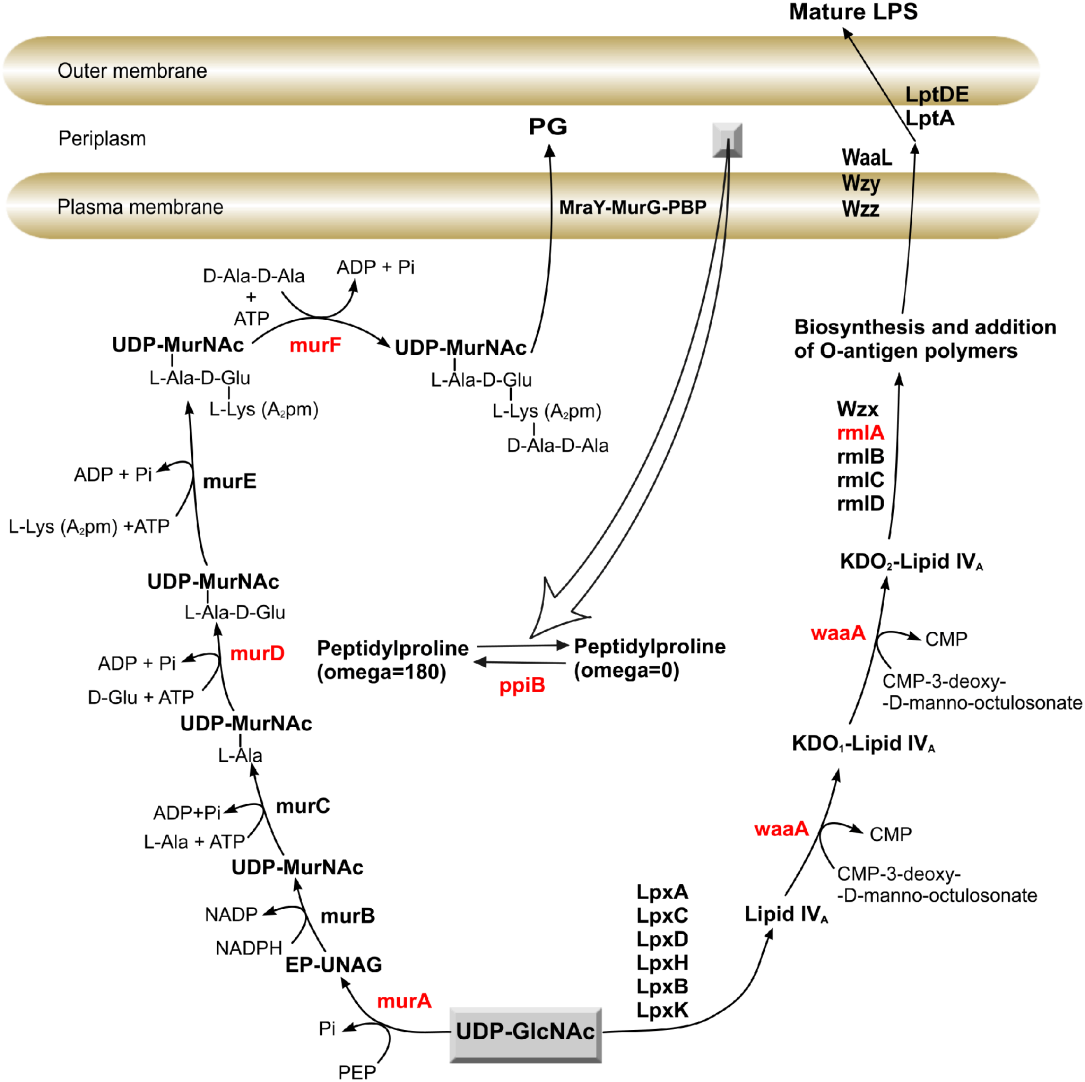
Schematic representation of peptidoglycan and LPS biosynthesis pathway in *P*. *aeruginosa*. The targeted genes are colored in red.

Most of *P*. *aeruginosa* strains develop high intrinsic resistance as a result of inefficient antibiotics uptake across the outer membrane [9]. A prominent constituent of the outer membrane is cell wall polymer, peptidoglycan, which is vital for bacterial survival. As in many other gram negative bacteria, the biosynthetic pathway of peptidoglycan consists of two-stage process. The first process takes place in the cytoplasm catalysed by murA. This is followed by reduction reactions of the enol-pyruvate moiety to D-lactate, yielding UDP-N-acetylmuramate catalysed by murB. The later product enters series of pentapeptide side chain additions on the newly reduced D-lactyl group which is processed by murC, murD, murE and murF (Fig 1) [10, 11].

The second process deals with the transfer of the precursor across the inner membrane and addition to the growing cell wall polymers [11]. Every antibiotic that is introduced into clinical use has limited or no effect on mur enzymes, except murA, which is inhibited by phosphonomycin [12]. Another promising target in *P*. *aeruginosa* cell-wall is the LPS biosynthesis pathway. The sugar 3-deoxy-D-manno-octulosonic acid (Kdo) is an essential component of the lipopolysaccharide (LPS) of *Escherichia coli* and other Gram-negative bacteria [13–15]. The Kdo sugar synthesis mediated by waaA (3-deoxy-D-manno-octulosonic-acid (Kdo) transferase) which makes sequential addition of two Kdo sugars onto a molecule of lipid IV_A_, a key precursor of lipid A (Fig 1). Belunis *et al*. [16] demonstrated that the 3-deoxy-D-manno-octulosonic-acid (Kdo) transferase gene is essential for growth of *E*. *coli*. Another LPS decoration is the O-specific polysaccharide, made of replicated oligosaccharide units. Most of the enzymes involved in the biosynthesis of O polysaccharides are encoded by genes clustered in the *rfb* locus (*rml* locus in *P*. *aeruginosa*) [17]. Mutations among *rfb* gene cluster in *E*. *coli* K-12 lead to loss of O antigen and inability to survive in its natural environment [18]. Moreover, previous studies have noted that the organism’s components residing in the periplasm are essential for *P*. *aeruginosa* pathogenesis and virulence [19]. Peptidyl-prolyl cis-trans isomerase B (ppiB), one of the most important enzymes in periplasmic area, which catalyses the cis-trans isomerization of proline peptide bonds, have been detected and isolated from both the periplasm of the *E*. *coli* and *P*. *aeruginosa* [19–21].

The lack of novel antibiotics against *P*. *aeruginosa* and related drug resistant Gram-negative bacteria is mainly responsible for the failure to control drug resistance [22, 23]. *P*. *aeruginosa* strain PAO1 has one of the largest bacterial genomes sequenced with over 5.500 predicted genes [24]. A multidisciplinary approach has been used to assess and validate drug targets, genetically and biochemically, characterise these targets and generate new hit [25, 26]. Based on bioinformatics and biological data assessment of PAO1 and other Gram-negative organisms, a target list of genes encoding proteins for which there are no structures and little biochemical data have been identified and predicted to have an essential function in this pathogen [25, 27].

The pathogenesis of *P*. *aeruginosa* infections is multifaceted, as illustrated by numerous virulence features. Previous studies have shown that the majority of bacterial outer leaflet murein, and LPS biosynthesis enzymes are crucial for the survival of most Gram-negative bacteria [11, 13–16, 18, 28]. However, no direct relationship between these genes and virulence of *P*. *aeruginosa* has been established. In order to fill the innovation gap and to search new targets for novel antibiotics development against *P*. *aeruginosa* infections, we used “evaluate and design” strategy. In this study, a sub-set of the PAO1 cell wall, murein and LPS biosynthesis targets were subjected to *in-vivo* gene deletion by constructing single-gene knockout strains of PAO1 followed by survival experiments of these mutants *in vitro* and *in vivo*. We report here the results obtained with a number of k.o mutants in *murA*, PA4450; *murD*, PA4414; *murF*, PA4416; *ppiB*, PA1793; *rmlA*, PA5163; *waaA*, PA4988. By using this genetic approach, we could assess directly the role of selected targets in pathogenesis and virulence in mice.

## Material and methods

### Ethics statement

All animal experiments were carried out according to recommendations of the European Commission and the law for the Care and Use of Laboratory Animals of the Government of the Federal Republic of Germany. The protocol and experiments were approved by the responsible German authorities (the Ethics Committee of Animal Experiments, The Landesuntersuchungsamt-the government of Rheinland-Pfalz, Federal Republic of Germany, Permit Number: 23 177-07/G09-15-001.

### Construction of suicide vectors for replacement of target genes

**Fig 2:**
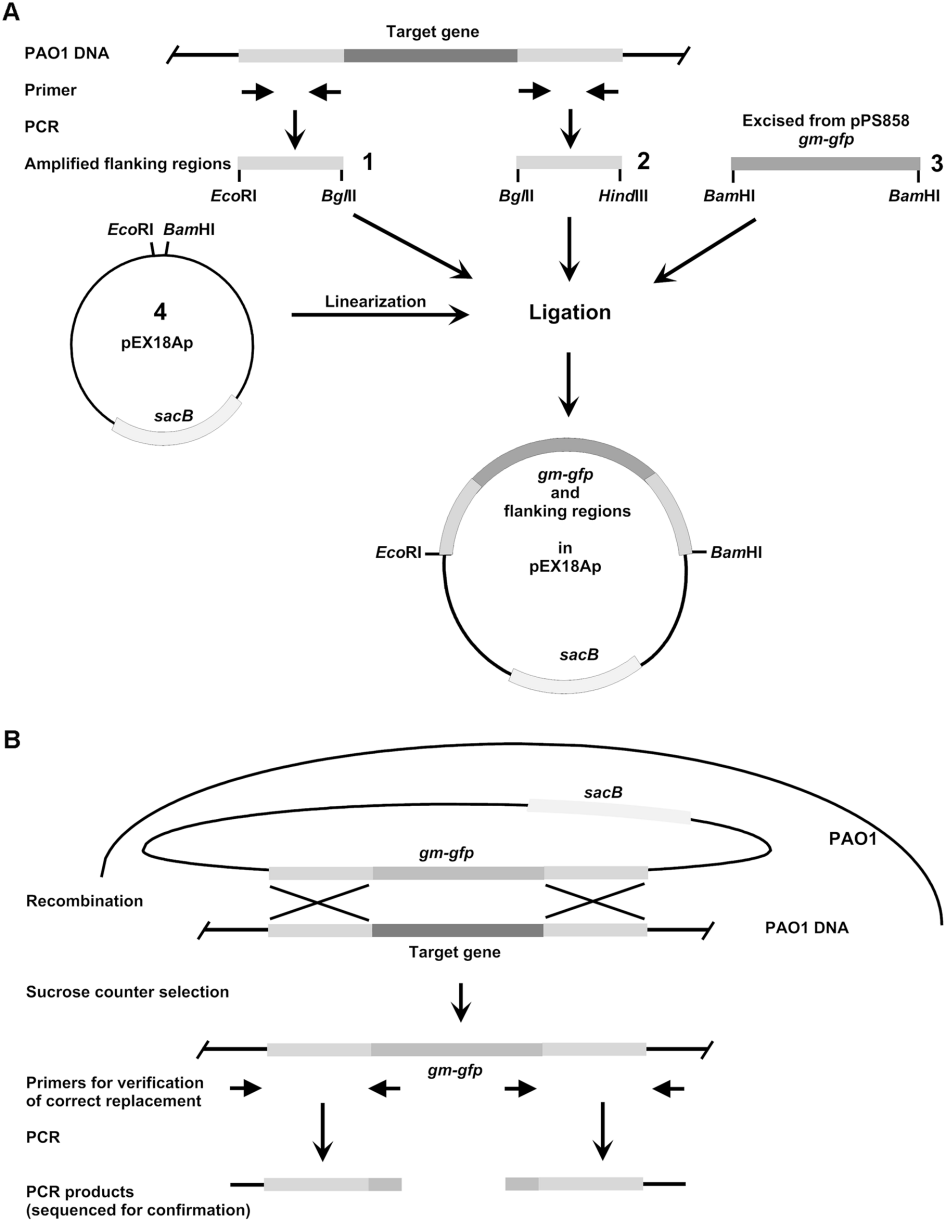
General scheme of construction of suicide vectors (A) and replacement of target genes (B). A: For construction of suicide vectors for replacement of target genes, regions of approximately 400 bp flanking the target up- and downstream, respectively, were amplified using primers with integrated restriction sites allowing directed insertion into mobilisable vector pEX18Ap. PCR-products of upstream regions (1) were cleaved with *Eco*RI and *Bgl*II and downstream regions (2) with *Hin*dIII and *Bgl*II (only in case of PA1793 *Bam*HI instead of *Bgl*II). The gentamycin-GFP cassette (3) of pPS858 was excised using *Bam*HI and vector pEX18Ap was linearised with *Eco*RI and *Hin*dIII. Cleaved fragments 1, 2, and 3 and linearised vector pEX18Ap (4) were then combined in ligation mixture. The gentamycin-GFP cassette ligation to up and downstream fragments aided by the compatible cohesive ends between *Bam*HI and *Bgl*II. Then *E*. *coli* donor strain ST18 was transformed with this mixture. Afterwards the suicide constructs were transferred from *E*. *coli* ST18 into *P*.*aeruginosa* PAO1 by conjugation. B: After conjugational transfer of suicide constructs recombinational replacement of the target genes and loss of vector backbone was forced by sucrose counter selection and gentamycin selection, respectively. Total DNA was isolated from knock out mutants and correct gene replacement was confirmed by sequencing extended PCR-products of the flanking regions.

For gene replacement the *sacB*-based strategy based on the pEX18Ap suicide vector [29, 30] was used. The suicide vectors for deletion of target genes were constructed by amplification of approximately 400 bp flanking regions using primers with integrated restriction sites (Table 1) allowing directed insertion into mobilisable vector pEX18Ap. PCR-products of upstream regions were cleaved with *Eco*RI and *Bgl*II and downstream regions with *Hin*dIII and *Bgl*II (only in case of PA1793 (*ppiB*) *Bam*HI instead of *Bgl*II). The gentamycin-GFP cassette of pPS858 was excised using *Bam*HI and vector pEX18Ap was digested with *Eco*RI and *Hin*dIII. Cleaved fragments and vector were then combined in ligation mixture (Fig 2). The constructs were confirmed by sequencing and then transformed into the *E*. *coli* donor strain ST18 which was used for conjugational transfer of the plasmids into *P*. *aeruginosa* PAO1 wild-type (WT) as described previously [29]. Briefly, *E*. *coli* ST18 containing the plasmid were streaked and transferred to LB plate containing aminolevulinic acid, carbenicillin 100 μg/mL and gentamicin 10 μg/mL. After controls and main cultures with and without antibiotics, in 1.5 mL reaction tube 100 μL PAO1 and 1 mL ST18 culture were mixed. The mixture was centrifuged at 11.000 g for 1 min, the pellet was suspended in 100 μL LB medium and plated on LB agar plate overnight at 37°C. Using an inoculation needle, the colonies from the conjugation plate transferred to LB-antibiotic plate and incubated overnight at 37°C. Single colonies obtained on the conjugation plate restreaked on LB-antibiotic agar plates. After counter-selection on LB-agar plates containing 5% sucrose and 80 μg/ml gentamycin, the obtained clones were tested for carbenicillin sensitivity by replica plating. In order to confirm the loss of plasmid borne DNA due to recombination events, genomic DNA was isolated from the potential k.o mutants and used in PCR together with primers specific for internal sequences of backbone of the plasmid. The correct replacement of the target gene by the gentamycin cassette was confirmed by site specific PCR and sequencing. Primers located in approximately 800 and 1400 base pairs distance from the ends of the cassette and directed outwards were used in combination with primers located minimum 500 base pairs up-and downstream from the target gene. The resulting PCR products were isolated and sequenced.

**Table 1:** Sequences of primers used for amplification of flanking regions of target genes, pEX18Ap vector backbone and Gm-gfp cassette.

Target gene	Flanking region	Primer sequence 5´-3´
PA1793 (*ppiB*)	upstream	GGCAATCGCCAGCGAATTC
		GCGACGAGATCTGTGGGTAATCCGCTTTGTC
	downstream	GCGACGGGATCCAGCGATGAGCGTCCTGTTC
		GCGACGAAGCTTCCAGCGGCGCAGGCGCATG
PA4414 (*murD*)	upstream	GGAGGAGAATTCGTACCTGCTGATTCCCAAC
		GGAGGAAGATCTGCTCTCTTCGTCCTCAACG
	downstream	GGAGGAAGATCTGATGCTGTCGGTGTTGCG
		GGAGGGAAGCTTCACGCAGACCTTGGCGATC
PA4416 (*murF*)	upstream	GGAGGAGAATTCCTGGAAAAAGTCCTGGAGG
		GGAGGAAGATCTGCGGCACCTCCCAGGCGGC
	downstream	GGAGGAAGATCTCTAATGCTCCTGCTGCTGG
		GGAGGGAAGCTTGACTGCCAGAAGTACTTCC
PA4450 (*murA)*	upstream	GGAGGAGAATTCGAGATCCTGCCTTTGCAGG
		GGAGGAAGATCTTGCAATGATCCCCCGTGGG
	downstream	GGAGGAAGATCTCGGAGGCTGTCGCGCAAATG
		GGAGGGAAGCTTCTTGATCACGTCGACCTGG
PA4988 (*waaA*)	upstream	GGAGGAGAATTCGAGATGGTCACGGTGTGCG
		GGAGGAAGATCTCATGGGCGCGCAGCTTAGC
	downstream	GGAGGAAGATCTGTAGCGCTCGCCCCGTCGG
		GGAGGGAAGCTTCGTGACCATCCAGTGGTTC
PA5163 (*rmlA*)	upstream	GGAGGAGAATTCCGACGAACTCAAGGTCGTG
		GGAGGAAGATCTGTGGTCCCTGCTCGCTCAG
	downstream	GGAGGAAGATCTGAAAGCGACCCGCCTGGC
		GGAGGGAAGCTTCCAGAAGTCGGTGGTCTTG
pEX18Ap vector	Fw	TATGTACTGTGTTAGCGG
	Rev	AAACTCTGGCTCACCG
Gm-gfp cassette	Fw	AACTTTGTATAGAGAGCCACTGCG
	Rev	TTAGGTGGCTCAAGTATGGGC

### Agarose bead preparation with *P*. *aeruginosa* PAO1 WT and *P*. *aeruginos*a PAO1 mutants

Agarose beads were prepared as described previously [31] with some modifications. *P*. *aeruginosa* PAO1 (ATCC 47085) wild-type (WT) was cultured overnight at 37°C in LB and cell wall targets mutants were cultivated in LB supplemented with gentamycin (80 μg/ml). After centrifugation at 1500 g for 10 min sedimented bacteria were resuspended in 1 ml sterile PBS (pH 7.4) and added to 5 ml 2% Agarose prewarmed to 50°C. The bacteria-agarose mixture was transferred rapidly to 5 ml 50°C prewarmed heavy white mineral oil. After intensive vortexing the mixture was cooled on ice for 5 minutes followed by centrifugation at 1500 g for 10 min. The resulting agarose beads were washed three times in sterile PBS (pH 7.4). The load of bacteria in the agarose beads was quantified by plating 10-fold serial dilutions on Columbia blood agar plates. The inoculums for infection in mice were prepared by diluting the bead suspension with PBS (pH 7.4) to 2x10^8^ CFU/ml.

### Lung infection of mice and quantification of bacteria in the lung

For the infection studies we used female NMRI outbred mice at the age of min. 6 weeks. The animals were anesthetized using a Ketamin-Xylazin mixture applied by subcutaneous injection. Mice were intratracheally infected with 50 μl of the bead suspension at a concentration of 2x10^8^ CFU/ml resulting in 1x10^7^ CFU. After 72 hours of infection, the mice were sacrificed by CO_2_ inhalation. Lungs were isolated and homogenized through cell strainer. Mouse lung homogenates were diluted 1:1, 1:10 and 1:100 with sterile PBS (pH 7.4). The resulted dilutions were plated on Columbia blood agar plates in case of *P*. *aeruginosa* PAO1 wild-type, and with addition of gentamycin (80 μg/ml) for Knockout mutants. The colony counts were determined for each dilution after overnight incubation at 37°C.

### Macrophage-mediated bactericidal assay

Macrophage-mediated bactericidal assays were carried out as described previously with few modifications [32]. The murine macrophage cell line J774A.1 (Leibniz-Institut DSMZ—Deutsche Sammlung von Mikroorganismen und Zellkulturen GmbH) was used to examine the survival *in vitro*. Macrophages were grown in DMEM supplemented with 10% fetal bovine serum (FBS) in a 5% CO_2_ atmosphere at 37°C. Mid-log-phase *P*. *aeruginosa* PAO1 wild-type grown in LB broth at 37°C and for mutants with addition of gentamycin were collected by centrifugation at 6.000 g and suspended to an OD_600_ of 0.4 in DMEM without FBS. Macrophages (5 × 10^7^) were incubated in 1 ml DMEM with the wild-type and mutants of *P*. *aeruginosa* (5 ×10^7^ CFU) for 30 min at 37°C. In order to eliminate extracellular *P*. *aeruginosa*, three washes by centrifugation at 163 g for 5 min at room temperature in PBS for wild-type and with PBS containing 400 μg/ml gentamycin were carried out. After the final wash, macrophages were allowed to adhere to tissue culture flasks in DMEM medium supplemented with gentamicin (400 μg/ml). After two hours of incubation, macrophages with internalized bacteria were harvested and lysed with 0.25% SDS and 0.025% SDS and the live intracellular bacteria were counted by plating serial dilutions of the lysates on LB plates for wild-type and LB containing gentamycin (80 μg/ml) for the mutants. The bacterial counts were determined with respect to the CFU/ml.

### Plant virulence assays

The plant virulence assay was performed in lettuce leafs as described previously [33]. 10 μl of stationary-phase cultures were diluted in LB medium (10^7^ CFU/ml; OD_600_ = 0.01), washed twice and resuspended in 10 mM MgSO_4_. Bacterial suspensions were inoculated into the midribs of Romaine lettuce leaves. Alu-dishes containing Whatman filter papers soaked with 10 mM MgSO_4_ and inoculated leaves were kept at room temperature for seven days. Symptoms were monitored daily for seven days.

### Culture conditions and growth curve

*P*. *aeruginosa* PAO1 was grown in LB medium or minimal M9 medium, if not otherwise stated. M9 medium contained 2% glucose as a carbon source in all cases. For growth of all PAO1 mutants both medium types were supplemented with gentamycin. For growth characteristics comparison, glycerol (2%), instead of glucose as a carbon source, was used.

### Determination of extracellular DNA

For the determination of the release of DNA by *P*. *aeruginosa* PAO1 cultures, the bacteria were grown in LB medium for up to 31 h. The cultures were centrifuged at 15.700 g for 10 min and the supernatant was mixed with loading buffer. The samples were subjected to 1% agarose gel stained with GelStar Nucleic Acid Gel Stain (Lonza, USA) and photographed under UV-light. Quantitative analysis of DNA on gel images was performed by ImageLab software (Bio-Rad Laboratories).

### Preparation for field emission scanning electron microscopy(FESEM)

*P*. *aeruginosa* PAO1 wild-type and the mutant strains were grown in LB and M9 medium. The media for k.o mutant were supplemented with gentamycin (80 μg/ml). 5 ml of midlog-phase cultures (OD_600_ of 0.7–0.9) were centrifuged and washed twice in PBS (pH 7.4). Samples were fixed with a solution containing 2.5% glutaraldehyde for 2 hours at room temperature and washed twice with PBS. Samples were further washed with TE buffer (20 mM TRIS, 2 mM EDTA, pH 6.9). After washing with TE buffer samples placed onto poly-l-lysine-coated slides and then dehydrated with 10%, 30%, 50%, 70%, 90%, 100% acetone, each step for 10 min on ice. The last 100% acetone step was performed at room temperature. Samples were then critical-point-dried with liquid CO_2_ (CPD 030, Bal-Tec, Liechtenstein) mounted onto aluminium stubs and sputter coated with gold (SCD 500, Bal-Tec, Liechtenstein) before examination in a Zeiss field emission scanning electron microscope Gemini DSM852 (Zeiss, Oberkochen, Germany) at an acceleration voltage of 5 kV using the Everhart-Thornley SE detector and the inlens SE detector in a 50:50 ratio. Images were stored onto a 230 MB MO disk. GIMP (version 2.0) software (http://www.gimp.org/) was used for images measurement.

## Results

### Construction of k.o mutants in *P*. *aeruginosa* PAO1

For validation of cell wall biosynthesis genes as potential new drug targets in *P*. *aeruginosa*, we created single gene knockout strains of *P*. *aeruginosa* PAO1 using *sacB*-based strategy on the pEX18Ap suicide vector (Fig 2). After the putative k.o mutants were identified by their carbenicillin-sensitive, gentamycin-resistant, and sucrose-tolerant phenotype, correct insertion of Gm-gfp cassette at the target site and excision of pEX18Ap vector backbone was confirmed. The latter event was examined by PCR using total DNA isolated from k.o-candidates together with primers specific to pEX18Ap vector backbone and Gm-gfp cassette (S1 Fig). In case where no fragment could be amplified, clones were further tested for replacement of target gene by Gm-gfp cassette. For this, primer combinations located minimum 500 bp from the flanking regions and those located in the cassette directed outwards were used. This means that the four primers combined in four pairs of which two were expected to result in a fragment in case of correct insertion. Amplified fragments were purified and sequenced for final confirmation.

### Growth characteristics of the k.o mutants: Δ*rmlA* failed to grow

**Fig 3:**
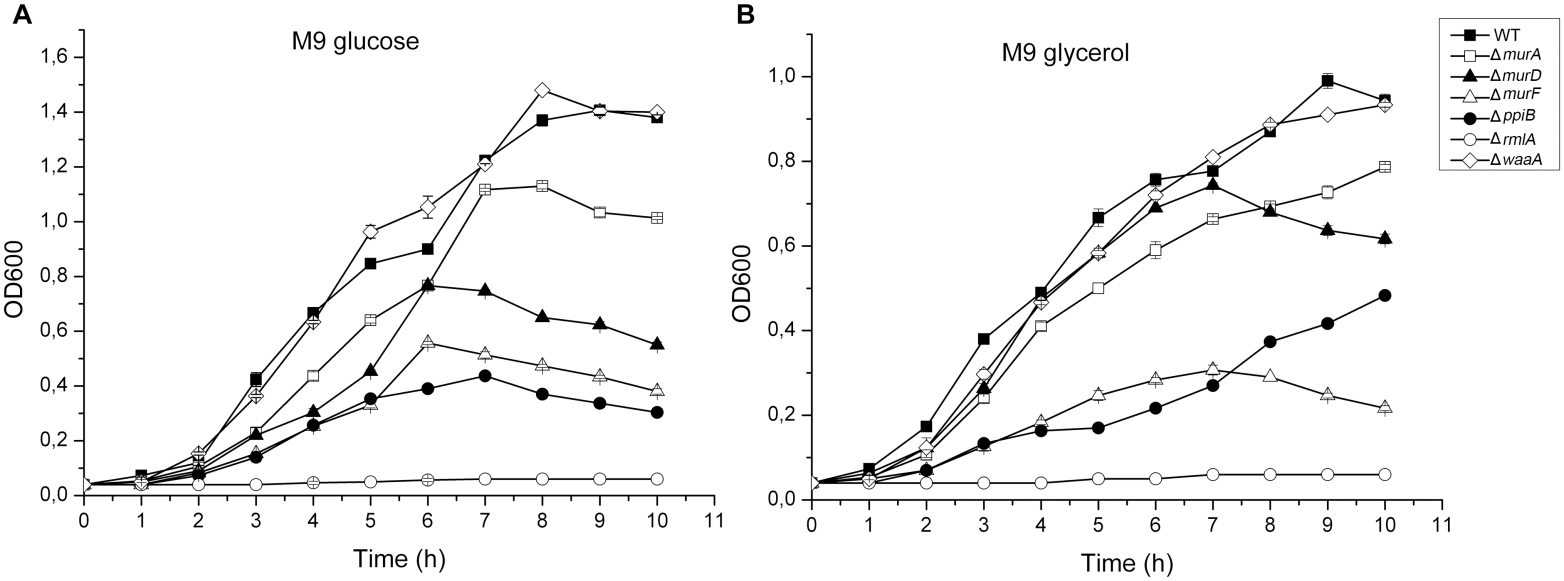
Growth characteristics of *P*. *aeruginosa* PAO1 wild-type and cell wall targets mutants. (A) Growth curves in M9 minimal medium with glucose as the sole carbon source. (B) Growth curves in M9 minimal medium with glycerol as sole carbon source. The standard errors of the mean for three independent experiments are shown.

Prior to studying the effect of the k.o mutations on the mice model, we first examined the growth characteristics of the wild-type and the mutant strains in both minimal and complex media. In liquid minimal medium M9 with glucose as the sole carbon source at 37°C, Δ*murA*, Δ*murD*, Δ*murF* and Δ*ppiB* mutant strains showed significantly reduced growth rate compared to that of the wild-type (Fig 3A). In minimal M9 medium with glycerol as carbon source, Δ*murA*, Δ*murD*, Δ*murF* and Δ*ppiB* mutant strains also showed significantly reduced growth rate compared to the parent strain (Fig 3B). Unexpectedly, Δ*rmlA* mutant strain failed totally to grow in M9 neither when supplemented with glucose nor with glycerol. The doubling time of all mutants except Δ*waaA* grown in the M9 medium either with glucose or glycerol was significantly longer compared to the wild-type strain. Unexpectedly the wild-type culture displayed short stationary-phase between 5 and 6 hours in M9 plus glucose and began to grow exponentially again after 6 hours. In glycerol supplemented media this short delayed growth appeared also between 6 and 7 hours. In addition, cultures of the mutant strains did not achieve the same final optical density as the parent strain in the M9 medium. In LB medium all mutants exhibited similar growth rate to wild-type except delayed growth in initial mid-log of Δ*rmlA* cultures (data not shown).

### Morphological and colony architectural changes

**Fig 4:**
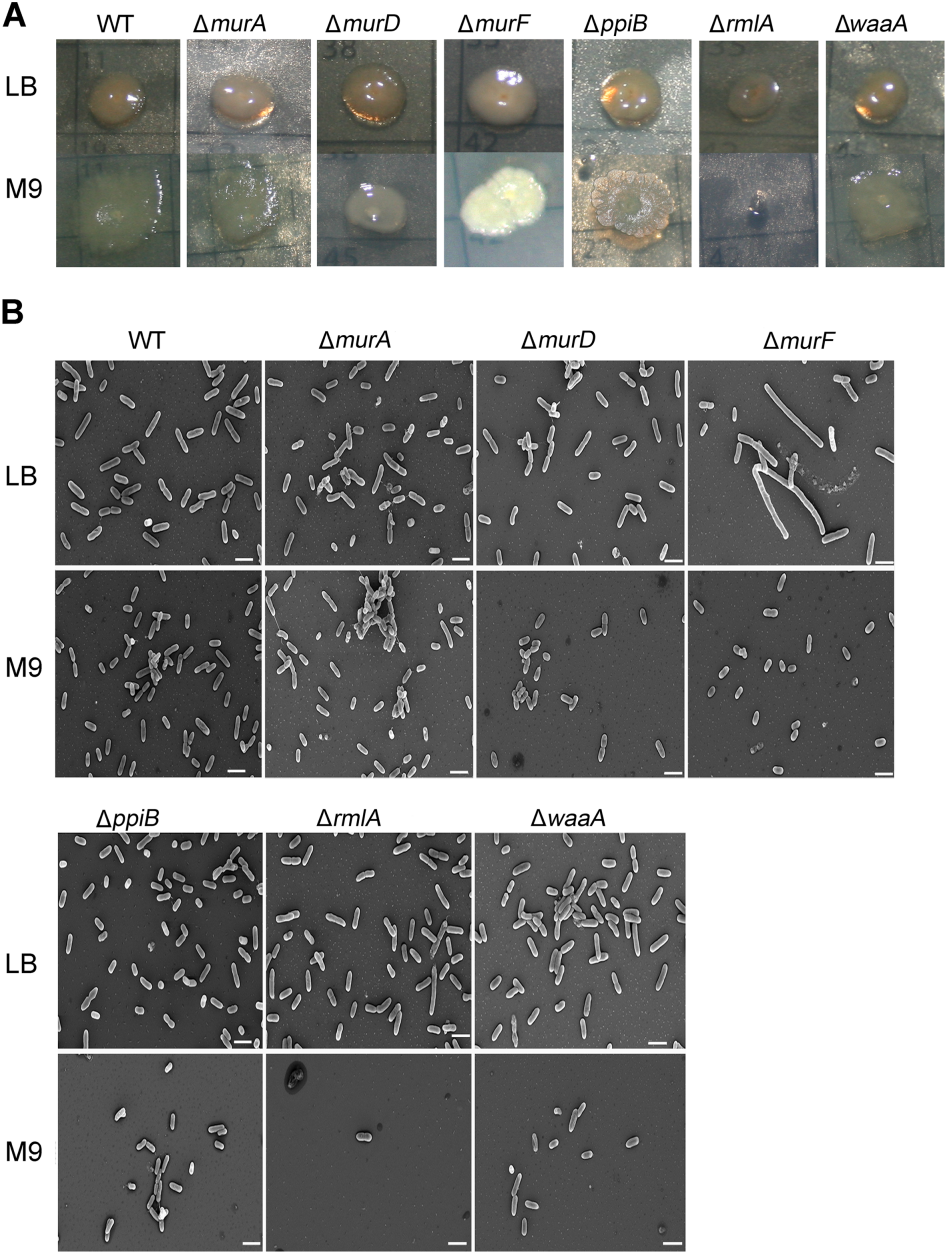
Morphological and colonial architectures changes in the knockout strains. (A) Colony morphology on LB (higher panel) and on M9 supplemented with glucose (Lower panel). (B) FESEM analysis of *P*. *aeruginosa* wild-type and cell wall mutants, all strains cultivated on LB (higher panel) and on M9 supplemented with glucose (Lower panel). Scale bar is indicated at the bottom of each image. The scale bars always represent 2 μm.

Using PAO1 k.o mutant cultures grown in minimal and complex media, we investigated colony morphology of PAO1 wild-type, Δ*murA*, Δ*murD*, Δ*murF*, Δ*ppiB*, Δ*rmlA* and Δ*waaA* grown *in vitro*. For colony morphology studies, single colonies were inoculated onto fresh medium plates (LB or M9 supplemented with glucose). All strains used in this study formed smooth colonies when cultured on LB. We noted that the Δ*murA* and Δ*murF* colonies in LB had different color with slightly shining and smoother textured surfaces (Fig 4A). On the other hand, dramatic changes in colonies morphology were observed when the wild-type and mutant strains were grown on M9 agar. All mutants colonies were smaller compared to wild-type except Δ*rmlA* strain which lost the ability to grow in minimal media (Fig 4A). Such effect in *rmlA* mutant (Fig 3) has also been reported for *E*. *coli* [18]. Δ*murA* strain showed similar undulated colonies like wild-type but were smaller in size. Δ*murD* formed flat smooth colonies with less undulations while Δ*waaA* formed lobed and smooth colonies. Moreover, Δ*murF* and Δ*ppiB* colonies had irregular and highly textured surfaces with marked wrinkles (Fig 4A). Taken together, the deletion of cell wall biosynthesis genes influenced the morphological appearance especially in minimal conditions. Scanning electron microscopy showed that whereas PAO1 wild-type grown in LB exhibited normal rod shape morphology with a cell length of 1.2–2.5 μm (Fig 4B), Δ*murA*, Δ*murD*, Δ*ppiB*, Δ*rmlA* and Δ*waaA* mutants grown in LB exhibited no alteration in cell morphology. Markedly Δ*murF* cells were highly elongated (Fig 4B) up to 6.8 μm compared to the wild-type and other mutants. This abnormal cell elongation is likely caused by defective cell division because most of elongated cells didn’t show any cell dividing pattern which may indicate that the formation of septa is suppressed in Δ*murF* mutants [34]. Thus, Δ*murF* may have an important role in septal formation and late cell division events [34, 35]. In M9 medium, length measurements demonstrated clearly that Δ*murD* and Δ*murF* cells are shorter when compared to wild-type and other mutants. For Δ*rmlA* strain in M9 very few cell found to be documented, because Δ*rmlA* strain lost growth ability in M9 medium and most probably these few cells were from the initial inoculum. These prominent and different changes in cell size, different colony morphologies and surface structure demonstrated that each Knockout gene affects the cellular phenotype differently.

### Extracellular DNA in *P*. *aeruginosa* cultures

**Fig 5:**
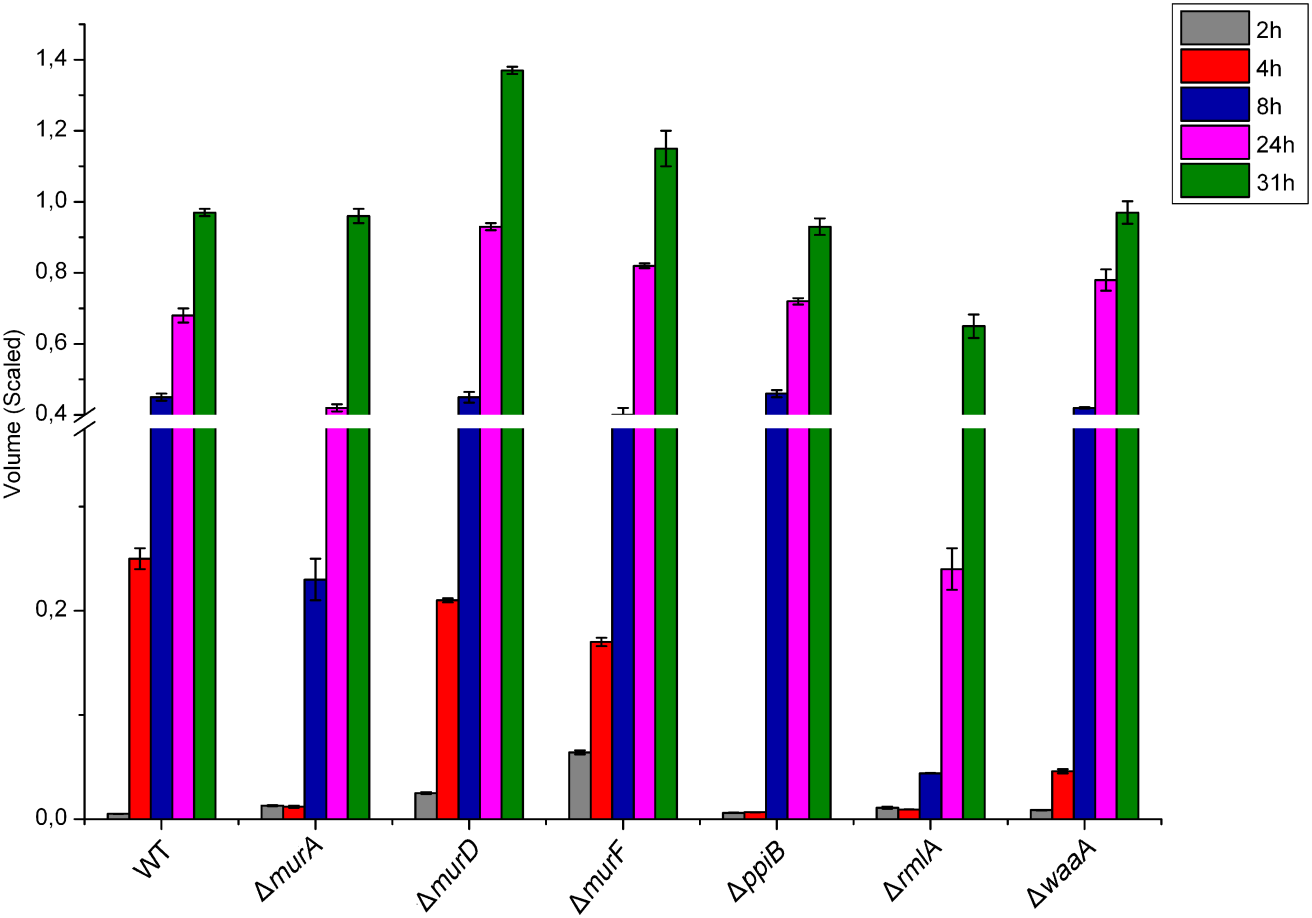
Cell wall genes dependent release of extracellular DNA. PAO1wild-type and cell wall targets mutants were grown in LB medium. Supernatant samples were collected after the respected time. DNA release was delayed and was much lower in the PAO1 *rmlA* mutants. Standard errors represent the mean of three independent experiments.

*P*. *aeruginosa* PAO1 as well as clinical *P*. *aeruginosa* isolates are known to release extracellular DNA [4, 36]. Accumulated evidence confirms that the extracellular DNA functions as a cell-to-cell interconnecting matrix component in biofilms [4, 36–38]. Moreover, extracellular DNA induces antibiotic resistance in biofilms [4]. The extracellular DNA in the medium of growing *P*. *aeruginosa* PAO1 strains was assessed by stained agarose gel electrophoresis. The LB medium was used because different growth rates were observed in minimal medium. Software-aided DNA density volume measurements, showed that the *P*. *aeruginosa* cultures except culture of Δ*murF*, contained a low basal level of extracellular DNA in the initial-log phase of growth (2 hours), and that a large amount of DNA was released in the late-log phase of growth (Fig 5). After 4 hours, sudden increase of extracellular DNA in wild-type, Δ*murD* and Δ*murF* cultures was observed, while the DNA amount remained low in the other mutants. This increase was followed by substantial increases of extracellular DNA after 8 hours in all except in Δ*rmlA* cultures. Obviously Δ*rmlA* cultures contained the lowest extracellular DNA among the whole time course. Fig 5 shows that a large amount of extracellular DNA was released specifically in the late-log phase of Δ*murD* growth. The low level of DNA release in Δ*rmlA* cultures correlated well with the delayed initial mid-log in LB and suggested that quorum sensing and biofilms might be affected seriously in this mutant [36–38].

### Cell wall mutants are sensitive to macrophage-mediated killing

**Fig 6:**
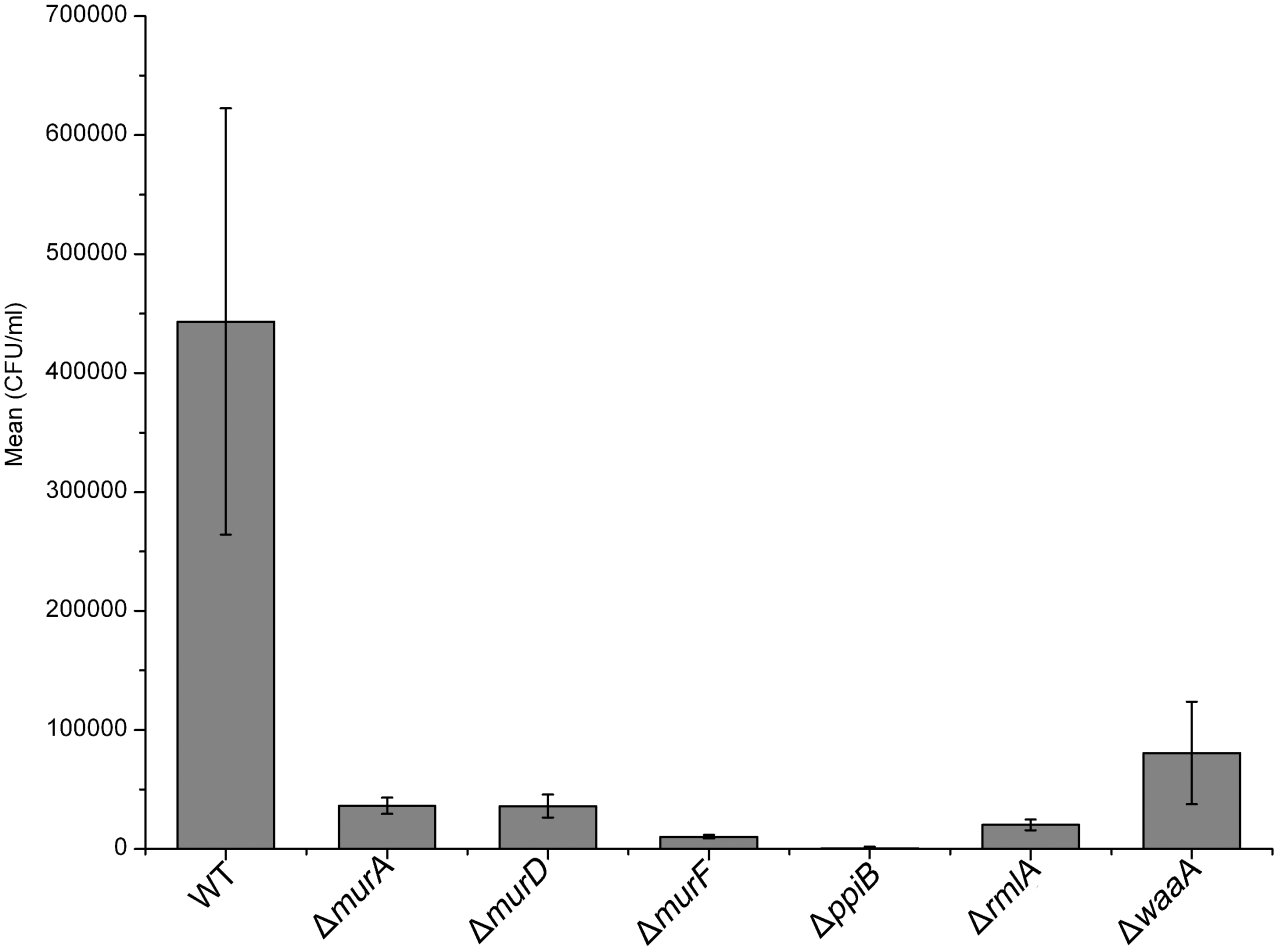
Macrophage-mediated bactericidal assay. Deletion of cell wall biosynthesis genes resulted in a significantly decreased survival of the bacteria. Standard errors of mean of 3 experimental points are shown.

On the basis of the association of outer leaflet murein, and LPS biosynthesis genes with virulence of most Gram-negative bacteria, we hypothesized that deletion of the these genes would lead to attenuation of intracellular macrophage growth [11, 13–16, 18, 28]. Encouraged by the growth rate decrease of most of the mutants in defined media, we measured the sensitivity of bacterial cells to macrophage-mediated killing. We performed macrophage cell culture invasion assay for both the wild-type and mutants using the murine J774 macrophage cell line *in vitro*. Macrophage cultures were infected with bacterial suspensions and intracellular bacteria were recovered in order to determine the number of surviving bacterial cells. As shown in Fig 6, all cell wall mutants were much more sensitive to macrophage-mediated killing than wild-type PAO1. These results suggested that cell wall biosynthesis genes are necessary for survival inside macrophages.

### Intratracheal lung infection of NMRI mice

**Fig 7:**
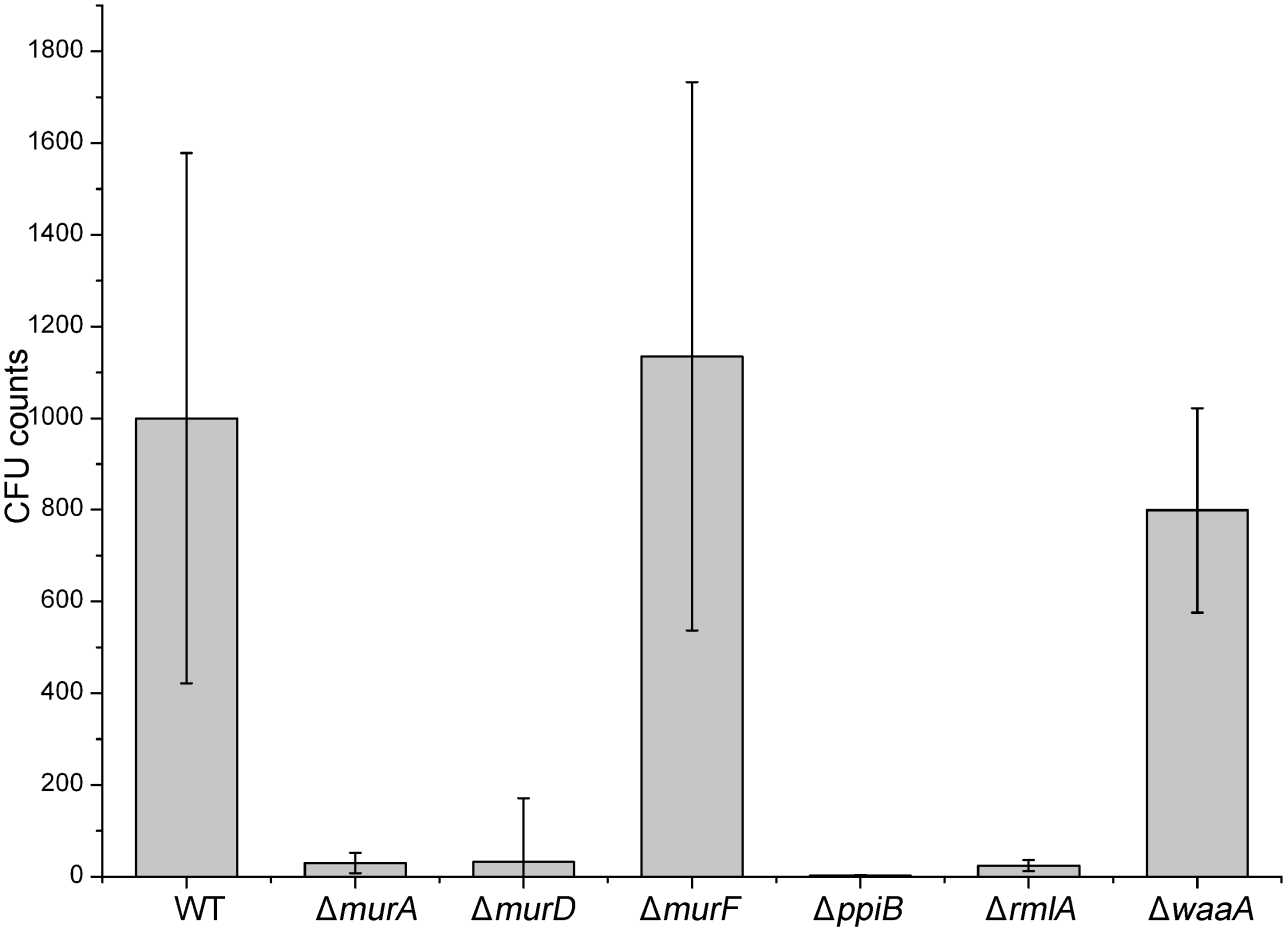
Assessment of the effect of cell wall targets gene deletion *in vivo*. Quantification of *P*. *aeruginosa* colonies grown in the lung of mice intratracheally infected with agarose beads loaded with the *P*. *aeruginosa* mutants in comparison to PAO1 wild-type (WT).

For further validation of outer cell wall biosynthesis genes as potential targets for novel drugs against *P*. *aeruginosa* infections, NMRI mice were intratracheally infected with agarose beads loaded with *P*. *aeruginosa* PAO1 and mutants as described in the method section. In our preliminary studies, incubation duration of 72 hours was found to yield the most significant results. Mice were separated in different groups of 10 animals each. 72 hours post infection the lungs were dissected and their homogenates were plated in different dilutions for quantification of the bacterial infectivity. In this study, *P*. *aeruginosa* PAO1 Δ*murF* and Δ*waaA* did not show any decrease in total bacterial counts compared to the wild-type (WT) strain (Fig 7). In contrast, Δ*murA*, Δ*murD*, Δ*ppiB* and Δ*rmlA* show reduced bacterial burden in mice. These results demonstrated that the *murA*, *murD*, *ppiB* and *rmlA* genes are required for infectivity and growth of *P*. *aeruginosa* in an *in vivo* model of infection while *murF* and *waaA* genes not.

### The k.o mutations in cell wall targets compromise *P*. *aeruginosa* in a plant virulence model

**Fig 8:**
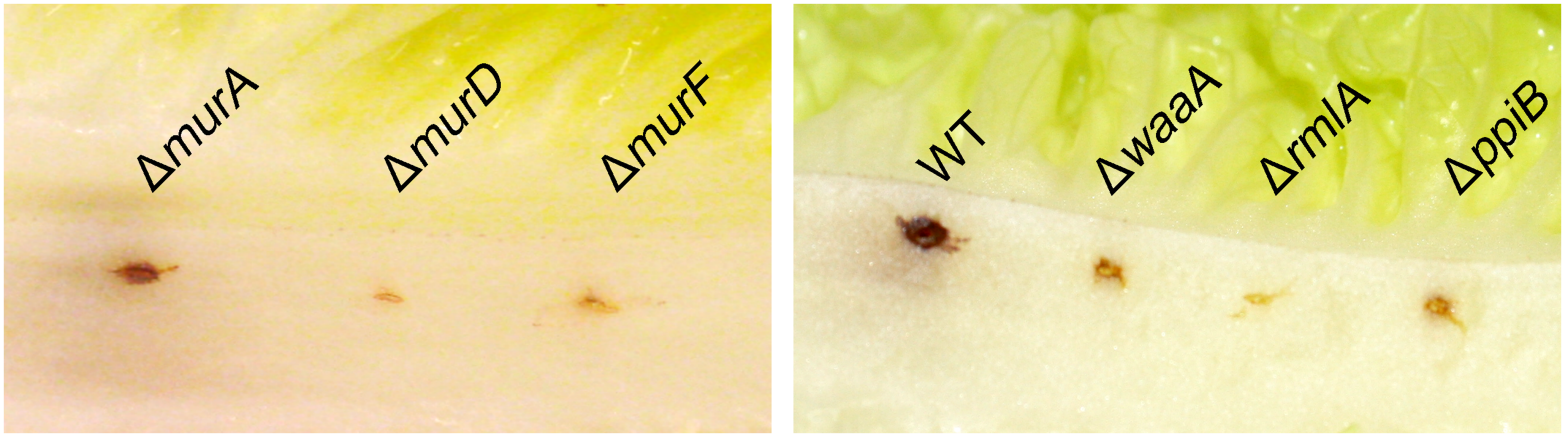
Plant (lettuce) virulence assay with the *P*. *aeruginosa* PAO1 and cell wall biosynthesis genes mutants. The figure represents lettuce midribs after 5 days of infection. Infection by PAO1 wild-type and *murA* mutant shows necrosis and tissue maceration. Three independent experiments gave similar results.

Apparently, the deletion of cell wall biosynthesis genes substantially decreased the *in vitro* intracellular survival in the murine macrophages and the *in vivo* survival in NMRI mice model of infection. For further assessment of the missing genes on the virulence of *P*. *aeruginosa*, we employed the lettuce leaf model of infection. Earlier studies used the plant as an *in vivo* model for identification of unknown *P*. *aeruginosa* virulence factors (genes) in mammalian pathogenesis [33, 39–41]. All these studies clearly demonstrated that the *P*. *aeruginosa* virulence mechanisms and factors are conserved between plant and animal models [40, 41]. Interestingly, the plant model disclosed a significant difference between the wild-type and mutants in infection symptoms. We found that lettuce was not susceptible to infection by Δ*murD*, Δ*murF* and Δ*rmlA* (Fig 8). These mutants strain did not cause any infection symptoms to the leaves even after a prolonged incubation period. In contrast, the wild-type, caused necrotic lesions just after three days post-infection. Δ*waaA* and Δ*ppiB* elicited delayed weak rotting symptoms on lettuce stems. Surprisingly, Δ*murA* proliferated in lettuce leaves and elicited disease symptoms similar to those elicited by wild-type (Fig 8). At day 7 of infection, Δ*murA P*. *aeruginosa* strain invaded the entire midrib of a lettuce leaf resulting in severe maceration (S2 Fig); the infection severity is even more than what was observed for wild-type.

## Discussion

Drug development against *P*. *aeruginosa* infections requires detailed information on the putative drug targets which are validated. Construction and characterisation of specific gene deletions of targets in the host strain is an excellent way for such validations. We constructed knockout mutants of six cell wall targets to investigate the essentiality of candidate genes. We employed a verified knockout system using pEX18Ap suicide vector as our successful tool for knockout [29, 30]. We were able to inactivate the cell wall targets at the bacterial chromosomal locus. In support of a role for cell wall biosynthesis and involvement in environmental versatility of *P*. *aeruginosa* [11–14, 16, 18–21, 24], the construction of the *P*. *aeruginosa murA*, *murD*, *murF*, *ppiB*, *rmlA* and *waaA* mutants resulted in dramatic changes in the growth properties in minimal media compared with the wild-type (Figs 3 and 4). Major changes in the size and morphology of the colonies were also observed when cultures were grown on solid media. Previously, cell elongation in *P*. *aeruginosa* was reported to be caused by nutrient deprivation and anaerobic respiration [34, 42]. It was postulated that bacteria elongate to increase their nutrient uptake and to respond to nitric oxide, a spontaneous byproduct of the anaerobic respiration. Remarkably, here we observed that Δ*murF* cells were highly elongated (Fig 4B) compared to the wild-type and other mutants. Unlike the previous reports, our results suggest that cell elongation could be a consequence of gene function loss and has significant effects on the physiological process, cell division and on formation of septal peptidoglycan [34, 35]. The issues underlying the short cell size exhibited by wild-type and mutants cells and their subsequent adaptation to growth on minimal medium have not been further clarified. In this study we have clearly demonstrated that knockout of cell wall targets strongly remodels the extracellular DNA release in LB media. Significantly, Δ*rmlA* released very low DNA amount which suggests that *rmlA* contributes to interbacterial signaling and multi-cellular development processes in Pseudomonas biofilms [4, 36–38].

Furthermore, our results also demonstrated the impaired ability of these mutants to replicate in murine macrophages. In this study, we tested the infectivity of PAO1 mutants in the lung infection model. Using this method we are able also to examine directly whether individual gene knockout in PAO1 has lethality effect in the PAO1 strain in the lung. With two major exceptions, the growth of these k.o mutants was severely diminished *in vivo* in mice compared to that of wild-type (Fig 7). This indicates that *P*. *aeruginosa* PAO1 Δ*murF* and Δ*waaA* do not constitute a regulator controlling *P*. *aeruginosa* growth-associated virulence, but the other targets do so. Interestingly, the *P*. *aeruginosa* PAO1 Δ*murF* and Δ*waaA* mutants were attenuated for intracellular macrophage survival.

Discernibly, several studies have shown that the *P*. *aeruginosa* virulence determinants are conserved within animal and plant models [33, 40, 41]. The mutant of *murA* proliferated in lettuce leaves and severely caused disease symptoms even more than the wild-type especially at late infection phase (S2 Fig). It is interesting to speculate that *murA* mutation was spontaneously complemented in the plant model. The peptidoglycan building stone, UDP-N-MurAc, forms from UDP-N-acetyl-D-glucosamine (UNAG) and phosphoenolpyruvic acid via murA reactions. murA catalyzes unusual reaction where the transfer of the enolpyruvyl moiety of phosphoenolpyruvic acid to the 3′-hydroxyl group of UNAG mediated through cleavage of C-O bond of PEP and not via addition-elimination process utilizing the high energy P-O bond [43, 44]. The shikimate pathway in plants contains AroA (5-enolpyruvylshikimate-3-phosphate synthase), the only enzyme known to catalyze the identical unusual reaction as murA [44, 45]. This finding indicates that the AroA enzyme from the plant might replace the function of murA. Other possible explanation is the abolition of the synthesis of UDP-N-MurAc, could be replaced by another similar sugar from the plant.

Understanding the details of aftereffect of the functional loss of targeted gene would lay the framework for a focused strategy of drug design. Future work could now be directed towards whole-genome effects of these mutants. Microarray-based comparative techniques could be applied to learn more about the interplay between these mutations and ecological versatility of this pathogen. The current report provides initial insights into the relationship between the targets studied and the cell and colony characteristics of *P*. *aeruginosa*. This study also provides definitive evidence that cell wall targets activity contributes to intramacrophage/mice survival and pathogenesis. Thus, these results identify peptidoglycan/LPS assembly in the *P*. *aeruginosa* cell wall as essential targets which are expected to significantly increase the probability of finding novel anti-pseudomonas therapeutic drugs.

## Supporting information

S1 Fig Genetic analysis by specific PCR to pEX18Ap vector backbone and Gm-gfp cassette. Knockout of the native genes in the PAO1 strain was verified by PCR analysis. Chromosomal DNA of mutant strains was used as templates in PCR reactions. Upper gel part): using internal specific primers for Gm-gfp cassette, the expected 425 bp. Which confirm the insertion of the Gm-gfp cassette at the chromosomal locus. Lower gel part): For these mutants the absence of pEX18Ap vector backbone was confirmed by using backbone specific primers (538 bp PCR Product). The PCR reactions were analyzed by gel-electrophoresis on 1% agarose gel. The first lane contains molecular size markers (GeneRuler 1 kb DNA Ladder, Thermo Scientific).
(PDF)

S2 Fig Plant (lettuce) infection assay with the *P*. *aeruginosa* PAO1 and *murA* mutant strain. The figure represents lettuce midribs after 6 and 7 days of infection. Infection by *murA* mutant shows severe necrosis/maceration the infection symptoms more than wild-type. Three independent experiments gave similar results.
(PDF)
