# Supplementary figures and images for: Novel drug targets in cell wall biosynthesis exploited by gene disruption in Pseudomonas aeruginosa
Source: PLoS One. 2017 Oct 18;12(10):e0186801. doi: 10.1371/journal.pone.0186801 (PMC5646862; doi:10.1371/journal.pone.0186801)

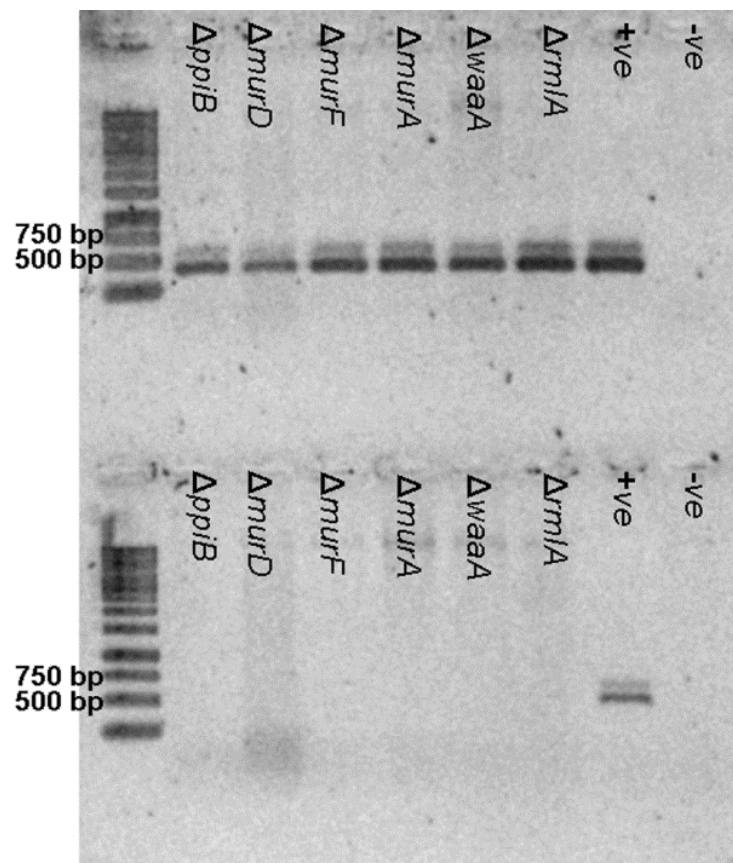

Supplement: S1 Fig — Knockout of the native genes in the PAO1 strain was verified by PCR analysis. Chromosomal DNA of mutant strains was used as templates in PCR reactions. Upper gel part): using internal specific primers for Gm-gfp cassette, the expected 425 bp. Which confirm the insertion of the Gm-gfp cassette at the chromosomal locus. Lower gel part): For these mutants the absence of pEX18Ap vector backbone was confirmed by using backbone specific primers (538 bp PCR Product). The PCR reactions were analyzed by gel-electrophoresis on 1% agarose gel. The first lane contains molecular size markers (GeneRuler 1 kb DNA Ladder, Thermo Scientific). (PDF) [file pone.0186801.s001.pdf]

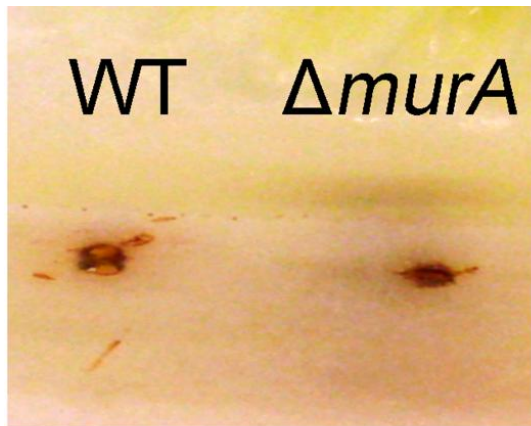

Day 6

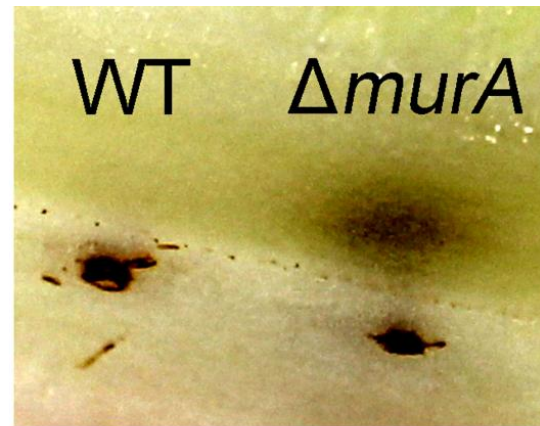

Day 7

Supplement: S2 Fig — The figure represents lettuce midribs after 6 and 7 days of infection. Infection by murA mutant shows severe necrosis/maceration the infection symptoms more than wild-type. Three independent experiments gave similar results. (PDF) [file pone.0186801.s002.pdf]
